# DNA binding with a minimal scaffold: structure–function analysis of Lig E DNA ligases

**DOI:** 10.1093/nar/gky622

**Published:** 2018-07-11

**Authors:** Adele Williamson, Miriam Grgic, Hanna-Kirsti S Leiros

**Affiliations:** Department of Chemistry, UiT The Arctic University of Norway, N-9037 Tromsø, Norway

## Abstract

DNA ligases join breaks in the phosphodiester backbone of DNA by catalysing the formation of bonds between opposing 5′P and 3′OH ends in an adenylation-dependent manner. Catalysis is accompanied by reorientation of two core domains to provide access to the active site for cofactor utilization and enable substrate binding and product release. The general paradigm is that DNA ligases engage their DNA substrate through complete encirclement of the duplex, completed by inter-domain kissing contacts via loops or additional domains. The recent structure of a minimal Lig E-type DNA ligase, however, implies it must use a different mechanism, as it lacks any domains or loops appending the catalytic core which could complete encirclement. In the present study, we have used a structure-guided mutagenesis approach to investigate the role of conserved regions in the Lig E proteins with respect to DNA binding. We report the structure of a Lig-E type DNA ligase bound to the nicked DNA-adenylate reaction intermediate, confirming that complete encirclement is unnecessary for substrate engagement. Biochemical and biophysical measurements of point mutants to residues implicated in binding highlight the importance of basic residues in the OB domain, and inter-domain contacts to the linker.

## INTRODUCTION

DNA ligases catalyze the formation of phosphodiester bonds between adjacent 5′P and 3′OH ends in the backbone of double-stranded DNA and have a conserved core structure comprising the catalytic adenylation (AD) domain and an oligonucleotide-binding (OB) domain joined by a flexible linker region (1). Catalysis proceeds in a three-step reaction, with step 1 involving nucleophilic attack, by a conserved lysine residue on the α-phosphate of an adenylated cofactor, which can be ATP or NAD+, generating a covalent enzyme-adenylate that is poised to bind the nicked DNA substrate. In step 2, the adenosine monophosphate (AMP) moiety is transferred to the 5′ phosphate of the nicked DNA activating it for attack by the 3′OH in step 3 forming a new phosphodiester bond that seals the break across the DNA backbone. This catalytic process is accompanied by large-scale reorientations of the AD and OB domains to expose the catalytic site for cofactor and substrate binding and clamp around the DNA during steps 2 and 3 of catalysis (1). In most cases, complete encirclement of the duplex is completed through participation of appending DNA binding domains, which are either N-terminal in the case of ATP-dependent DNA ligases or C-terminal in the NAD-dependent form (2,3). Alternately, smaller viral ligases for example the Chlorella virus (ChlV-Lig) and presumably T7 ATP-dependent DNA ligases use flexible loops which become ordered upon DNA interaction (4). In some cases additional subunits are required for optimal activity, such as the Ku end-binding protein which activate the non-homologous end-joining bacterial ligase Lig D (5,6). For the larger ligases, this DNA binding domain is indispensable for productive substrate binding and ligase activity (7,8). Likewise, mutation or deletion of the Chlorella virus ligase latch decreases DNA affinity and activity (4,9).

The minimal Lig E-type DNA ligases comprise a clade of phylogenetically distinct ATP-dependent DNA ligases found almost exclusively in proteobacteria (10). The majority of these enzymes lack any appending domains and possess a predicted N-terminal signal sequence for localization in the periplasm, the removal of which increases both protein stability and activity (11). The recently-determined crystal structure of the enzyme-adenylate of Lig E from *Psychromonas* sp. strain SP041 (Psy-Lig) revealed a compact structure with no flexible loops or other elements that would be sufficient to surround the circumference of the DNA helix (12). Despite lacking the known binding determinants of larger ATP-dependent DNA ligases, Psy-Lig and other Lig Es from *Neisseria meningitidis*, and *Haemophilus influenza* are able to seal phosphorylated double-strand DNA substrates at comparable rates to their larger counterparts (12–14). Analysis of the Psy-Lig structure suggests it may engage its DNA substrate using well-ordered residues on the DNA-binding faces of the AD and OB domain. Modelling studies and sequence alignment indicate several highly-conserved basic residues on the surface of the OB domains that are positioned to interact with the DNA (12). In addition, potentially important inter-domain contacts have been identified in the structure which could facilitate binding by pre-arranging the domains in a favourable orientation (12).

To further explore the mechanism of Lig E DNA ligases we have carried out site-directed mutagenesis of Psy-Lig and determined the structure of a close homolog from the marine gamma proteobacterium *Alteromonas mediterranea* Lig E (Ame-Lig) bound to nicked adenylated DNA. The Lig E class of ATP-dependent DNA ligases represent the most minimal DNA ligases characterized to date, and as such, provide a simplified model to study the structure-activity relationship of these enzymes; information that may be especially useful information for guiding protein engineering endeavours.

## MATERIALS AND METHODS

### Cloning and expression

Wild-type (WT) *ame-lig*, the triple *ame-lig 3xmut* (K/Q/R-loop mutant) and quadruple *psy-lig OB-Ala* mutant were ordered as codon-optimized synthetic constructs from the Thermofisher GeneArt service. In the case of *ame-lig* the gene sequence encoded the predicted mature form of Lig E of *A. mediterranea* (WP_020742910) where a signal peptide with a predicted cleavage site between positions 21 and 22 was omitted from the sequence. The *psy-lig OB-*Ala construct was identical to the Psy-Lig WT (12) with the exception of point mutations described below. Synthetic genes were supplied sub-cloned into pDONR221 and were transferred into the pDEST17 plasmid (Invitrogen) as described previously (12). Site-directed mutagenesis of Psy-Lig to create single and double mutants was carried out on the pDONR221 construct using the Stratagene kit Quickchange II kit (Agilent) and the oligonucleotides listed in Supplementary Table S1. The Ame-Lig loop deletion mutant (Δ-Loop) was generated by amplification of pDONR221::*ame-lig* using primers listed Supplementary Table S1 to generate *ame-lig* 5′ and 3′ fragments excluding the loop-encoding region, and generating a new BamHI site at the junction. Digestion and ligation using BamHI and T4 DNA ligase (New England Biolabs) yielded the Δ-Loop *ame-lig* fragment. WT *ame-lig* was excised from pDONR221::*ame-lig* through the pDONR221 ApaI site and *ame-lig* HindIII sites, and the Δ-Loop *ame-lig* fragment ligated into the gel-purified backbone. Mutations made in pDONR221 constructs, were verified by Sanger sequencing with M13 primers before exchange into expression vectors. Protein expression and purification was carried out as previously described (11,12).

### Crystallization, structure determination and analysis

Single oligonucleotides listed in Supplementary Table S1 were resuspended at 9 mM in annealing buffer (50 mM Tris pH 8.0, 50 mM NaCl, 1 mM EDTA), mixed 1:1:1 to give a final duplex concentration of 3 mM and incubated at 85°C before cooling overnight. Ame-Lig (275 μM) was incubated with 1.2 molar equivalents of nicked duplex and 5 mM additional EDTA for 30 minutes on ice to form the protein-DNA complex. Cubic crystals approximately 0.05 × 0.05 × 0.05 mm^3^ were grown by hanging drop diffusion method at 4°C from 24% PEG 4K, 100 mM Bis-Tris pH 5.5; and appeared within 1 week. Crystals were cryoprotected in 24% PEG 4K, 100 mM Bis-Tris pH 5.5, 12% ethyleneglycol and flash frozen in liquid nitrogen. Diffraction data to 2.3 Å was measured at BL14.1, BESSY II, Berlin. Data was integrated, scaled and truncated in XDS, XSCALE (15) and AIMLESS (16). The complex structure was solved by molecular replacement using Phaser-MR (17) with chlorella virus DNA-protein complex and Psy-Lig enzyme-adenylate as search models, and further refined in COOT (18). Data collection and statistics are listed in Table 1.

**Table 1.** Data collection and refinement statistics for Ame-Lig bound to nicked DNA-adenylate (PDB entry 6GDR)

Resolution range (Å)	24.84–2.327 (2.41–2.327
Wavelength (Å), Beamline	0.9184; Bessy BL14.1
Space group	I 1 2 1
Unit cell: *a, b, c* (Å); β (°)	65.02, 71.21, 117.16; 94.82
Total reflections	156 848 (15 074)
Unique reflections	22 722 (2212)
Multiplicity	6.9 (6.9)
Completeness (%)	98.55 (96.09)
Mean 〈*I*/σ_(_*_I_*_)_?	14.6 (1.2)
Wilson B-factor (Å^2^)	55.29
*R*-merge	0.075 (1.387)
*R*-meas	0.088 (1.635)
*R*-pim	0.046 (0.858)
CC _1/2_	0.999 (0.493)
Resolution range in refinement (Å)	24.84–2.33 (2.41–2.33)
Reflections used in refinement	22 708 (2213)
Reflections used for *R*-free	1143 (118)
*R*-work	0.2180 (0.3053)
*R*-free	0.2641 (0.3155)
Number of: non-hydrogen atoms	3006
Protein/DNA	2049/856
Ligands (1 AMP, 2 PO_4_^2−^)	24
solvent	69
Number of protein residues	256
RMSD bonds (Å)	0.003
RMSD angles (°)	0.58
Ramachandran favoured (%)	94.80
Ramachandran allowed (%)	4.00
Ramachandran outliers (%)	1.20
Rotamer outliers (%)	0.47
Clashscore	2.03
Average B-factor all atoms(Å^2^):	64.15
Protein/DNA (Å^2^)	65.7/63.4
Ligands (Å^2^) (1 AMP, 2 PO_4_^2−^)	57.60
Solvent (Å^2^)	55.57

Statistics for the highest-resolution shell are shown in parentheses.

Structural comparisons were done using the PDBeFold server with default settings (19). Protein–DNA and inter-domain interactions were detected using the NuProPlot program (20) and the RING server (21) respectively.

### Ligation assays

Ligase activity of Ame-Lig and Psy-Lig mutants were measured by molecular beacon (MB) assay as previously described (12,22). Unless otherwise stated, the reaction conditions were 2.0 nM enzyme, 300 nM substrate, 0.1 mM ATP, 10 mM MgCl_2_, 1.0 mM 1,4-dithiothreitol (DTT), 100 mM NaCl, 50 mM Tris pH 8.0 at 30°C.

### Microscale thermophoresis binding assays (MST)

The fluorescently-labelled 40-mer nicked substrate was made by annealing 80 nM of FAM-labelled 3′OH oligo with a 5-fold excess of complement and 5′phosphate oligos in 50 mM Tris, 100 mM NaCl, 1.0 mM DTT. Oligos are listed in Supplementary Table S1. The solution was incubated at 85°C for 5 min and cooled to 25°C before use. Psy-Lig mutants were titrated into the DNA substrate over a concentration range of 0.1–10 μM and incubated at 4°C for 30 min prior to measurement. The final reaction mixture contained 40 nM nicked DNA substrate, 5.0 mM EDTA, 1.0 mM ATP, 0.5% Tween, 50 mM Tris, 100 mM NaCl, 1.0 mM DTT. MST analysis was carried out on the Monolith NT.115Pico using standard capillaries with instrument settings MST power 40%, excitation power 1%, temperature 25°C.

### Electrophoretic mobility shift assays (EMSA)

DNA binding by Psy-Lig mutants was measured by EMSA using the 18-mer singly-nicked substrate described previously (12) or a 40-mer linear substrate prepared as described above and listed in Supplementary Table S1. 80 nM substrate was incubated with a 40×, 20× or 10× molar excess of enzyme, 0.1 mM ATP, 50 mM Tris pH 8.0, 50 mM NaCl, 5.0 mM ethylenediaminetetraacetic acid (EDTA) at 15°C for 30 min. Samples were electrophoresed through an 8% polyacrylamide gel and visualized on a Pharox FX Plus imager (Biorad).

### Differential scanning fluorimetry (DSF)

DSF measurements for thermal stability of Psy-Lig mutants in the final purification buffer (50 mM Tris pH 8.0, 200 mM NaCl, 1.0 mM DTT, 5% glycerol) were carried out as described previously (23) with final protein concentrations of 10 μM (standard) or 2 μM (with DNA). Unfolding between 5 and 90°C was measured in the presence of: no additives; 100–500 mM NaCl; 0.1 mM ATP; 10 mM MgCl_2_ or DNA. The latter condition used double-stranded nicked MB substrate without the TAMRA (5-carboxytetramethylrhodamine) fluorophore or DABCYL (*N*-[4-(4-dimethylamino)phenylazo]benzoic acid) quencher and was added at an equimolar concentration to the protein together with 5 mM EDTA and 0.1 mM ATP.

### Circular dichroism (CD) spectrometry

The recombinant proteins were dialyzed overnight at 4°C against CD-buffer (10 mM Tris pH 8.0 and 100 mM NaF). The samples were filtered through a 0.45 μm pore size filter (Spin X Costar) to remove precipitate and diluted to a final concentration of 0.15 mg/ml. Data was collected on a J-810 CD spectrophotometer (Jasco) at 25°C using a 1 mm path length cuvette using the following settings: sensitivity 100 mdeg, datapitch 0.5 nm, scan speed 50 nm/min, response 2.0 s, bandwidth 1 nm, accumulation three scans, units CD mdeg. Three sample scans were recorded and averaged. Three scans of buffer were also recorded, averaged and subtracted.

## RESULTS

### Lig E partially encircles nicked DNA

**Figure 1.**
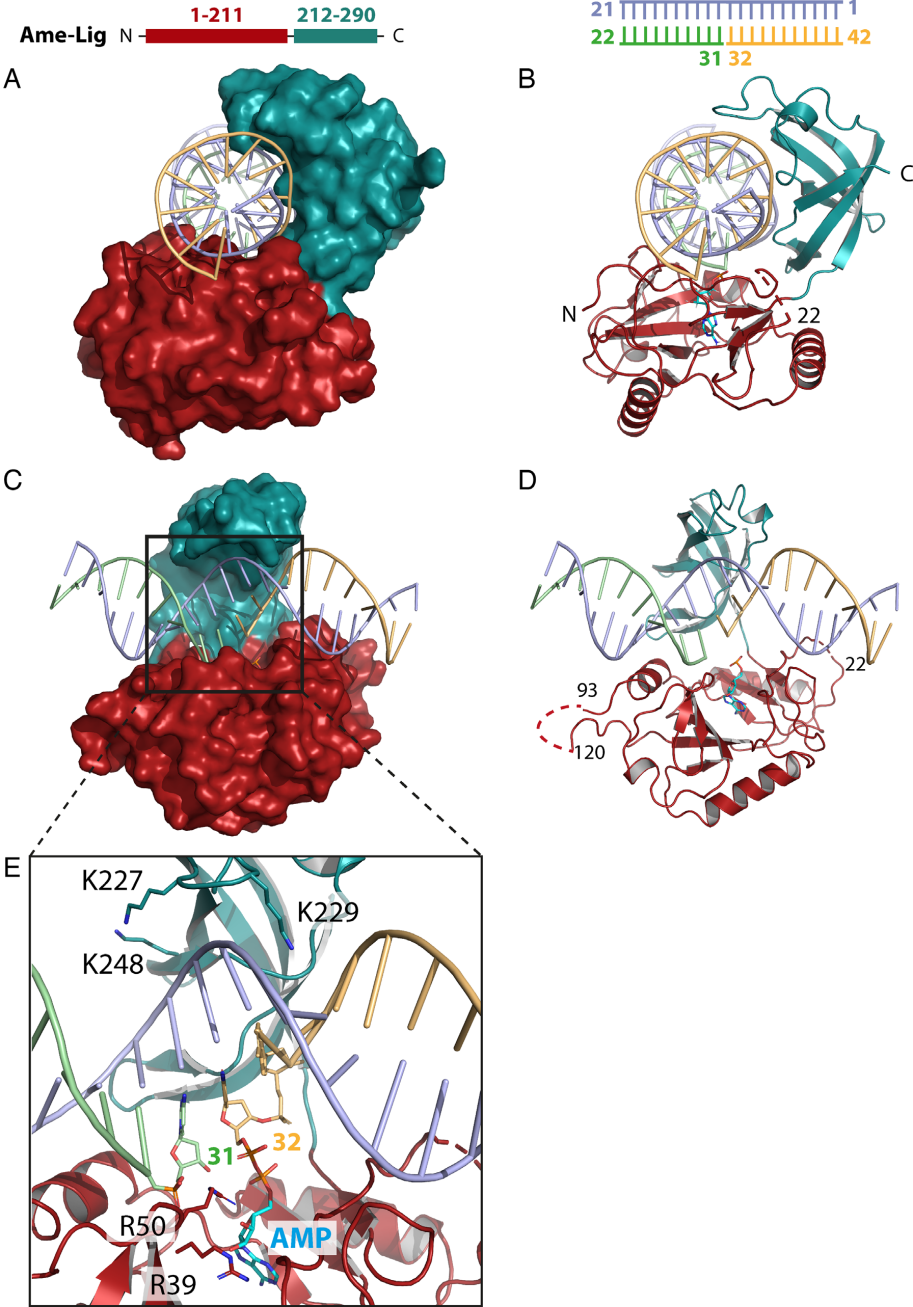
Two views of the overall structure of Ame-Lig bound to 21-bp nicked DNA-adenylate with the protein represented as a surface (**A** and **C**) or cartoon showing secondary structural elements (**B** and **D**). Detail of the active site is shown in panel **E**.

The overall structure of the Ame-Lig-DNA complex resolved to 2.3 Å clearly reveals that this Lig E-type ligase binds its substrate without complete encirclement of the DNA duplex (Figure 1). The complex contains one monomer in the asymmetric unit with the ends of the DNA engaged in crystal contacts forming a DNA filament through the crystal. As with other DNA-ligase intermediates, the core catalytic domains of Ame-Lig are oriented to form a C-shaped clamp about the DNA with the nick positioned above the AMP-binding pocket. Inspection of the electron density reveals a phosphodiester bond between the 5′P of the nicked strand and the alpha phosphate of the AMP, indicating that the step two intermediate has been crystalized after transfer of the cofactor from the catalytic lysine K34 to the DNA (Supplementary Figure S1). DNA encirclement by Ame-Lig encompasses ∼245° of the helix circumference, and the interaction buries 3837 Å^2^ of contact surface.

Continuous electron density is observed for most the protein chain from I7 to R290 with the exception of residues 20–21, and a 26 residue section in the AD domain (S94–S119). This section, located between helices α3 and α4, is presumed to form an unstructured loop. The importance of this region, which is absent in most other Lig Es, was tested by mutating three positively-charged residues (K91, Q98, R106) to alanine (hereafter Ame-Lig 3x mut) or replacing the non-conserved section of loop (L98-T115) with the truncated sequence G–S–G (Supplementary Figure S2A and B). Both variants had equivalent activity to the WT, indicating that this region is dispensable for regular ligation activity of Ame-Lig (Supplementary Figure S2C). Comparisons of structural homology made by superposition of individual domains indicates the Ame-Lig has similarity with Psy-Lig enzyme-adenylate (Table 2), indicating that comparison of these structures gives a good indication of variations between the DNA-bound and apo forms of Lig E.

**Table 2.** Structural alignment between Ame-Lig and other ligases that contain only the catalytic core domains; Psy-Lig, PDB 4D05; ChlV-Lig, 2Q2T, T7 DNA ligase, 1A0I. The domain boundary is between first lysine of motif V and the next residue. *Q*-score, quality score of the Ca-alignment between 1 and 0 with 1 being the highest score; *Z*-score, significance of the alignment based on Gaussian statistics; root-mean-square deviation (R.M.S.D.) between C-α atoms in the three-dimensional alignment

	*Q*-score (*Z*-score/R.M.S.D)	
Protein	AD-domain	OB-domain
Psy-Lig (A)	0.81 (14.52/1.07)	0.86 (10.08/0.81)
Psy-Lig (B)	0.83 (14.07/0.98)	0.89 (15.13/0.86)
ChlV (DNA-bound)	0.54 (9.91/2.01)	0.58 (7.24/0.71)
T7 DNA ligase	0.40 (6.84/2.02)	0.46 (6.79/1.76)

### Interactions of Lig E with DNA

**Figure 2.**
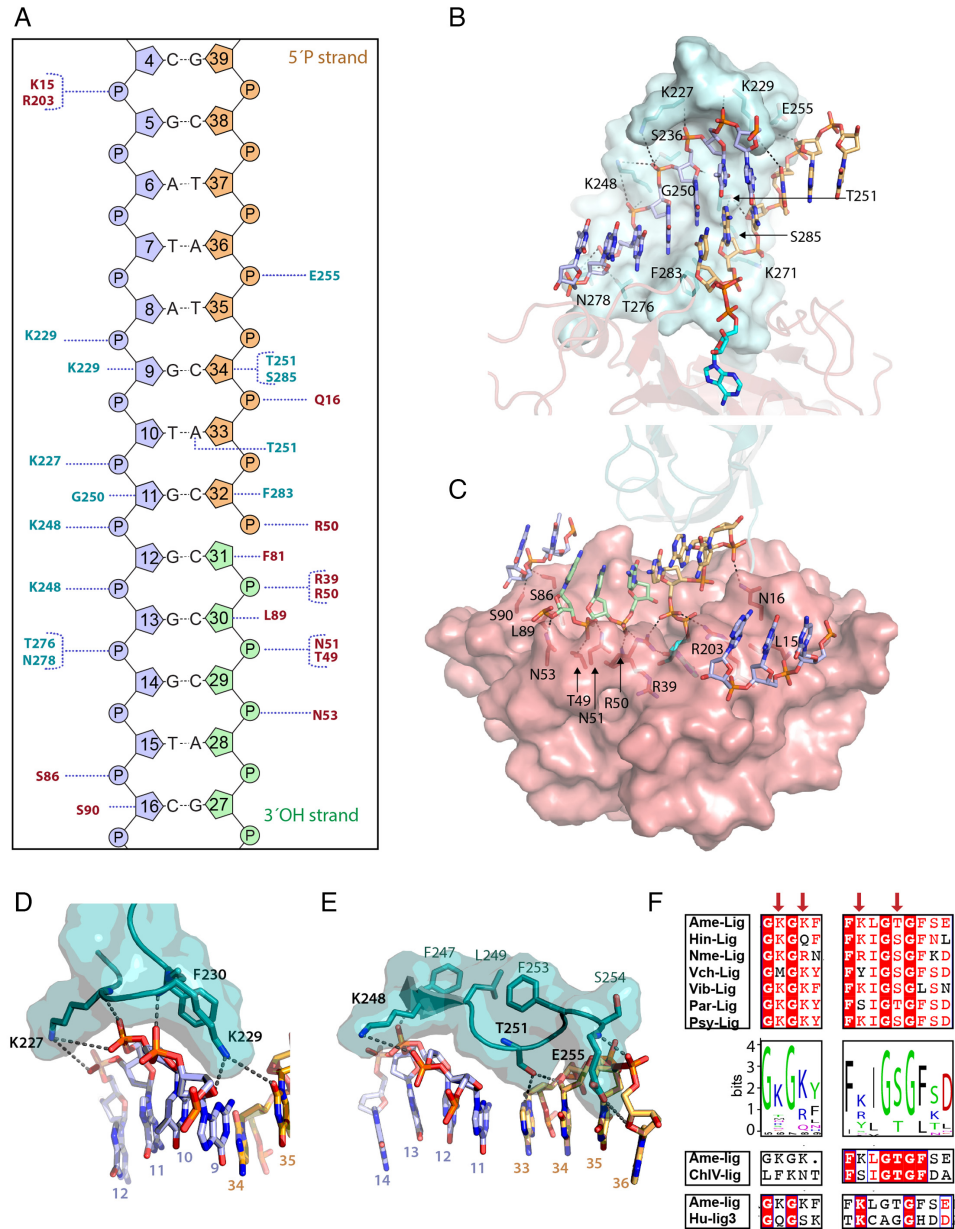
Ame-Lig binding to DNA. Throughout the figure the text and structure are coloured as follows: the AD-domain red and the OB-domain cyan, the 3′ OH oligo green, the 5′P oligo orange and the complement strand purple. Polar contacts are indicted by dashed grey lines. (**A**) Schematic of interactions for Ame-Lig predicted by NuProPlot (20). Default settings were applied (maximum distance 3.25 Å, Van der Waals distance 3.80 Å, minimum angle 85°) and confirmed by manual inspection. (**B**) Interactions of the complement and 5′PO_4_ strand with the OB domain. (**C**) Interactions of the nicked and complement strands with residues in the AD domain. (**D**) DNA interactions of conserved binding motif GKGKF. (**E**) DNA interactions with conserved motif KLGTG. (**F**) Conservation of motifs among a selection of aligned Lig Es (right) and sequence logo built from 542 Lig Es (Supplementary Table S2). Corresponding regions of Chlorella virus ligase (ChlV-Lig) and Human Lig 3 (Hu-Lig3) are shown for reference. Naming of sequences is as follows: Hin-lig, Haemophilus influenzae; Nme-lig, Neisseria meningitidis; Vch-lig, Vibrio cholera; Vib-lig, Aliivibrio salomicida; ChlV, Chlorella virus; Hu-lig3, Human ligase3.

The DNA-binding footprint of Ame-Lig is 8 nucleotides (nt29–nt36) on the nicked strand and 12 nucleotides on the complement (nt5–nt16), thus smaller than any previously-described DNA-bound ligase structure (Figure 2A). Notably, several positions within these margins are not contacted by Ame-Lig. This includes a three nucleotide stretch between nt6 and nt8 which is on the exposed face of the helix not encircled by the core domains as well as nt10 and nt15 of the complement and nt35 of the 5′nick strand. Both Ame-Lig and Psy-Lig are fully active and able to ligate a range of DNA breaks including single nicks, 4 base-pair cohesive ends and mis-matches at the nick site, and to a lesser extend gaps, although little or no activity is observed with blunt substrates for Psy-Lig and Ame-Lig respectively ((12) and Supplementary Figure S3A). This indicates that, despite lacking the extensive DNA circumferential engagement or extensive contact surface of larger DNA ligases, the minimal Lig E class of ATP-dependent DNA ligases are able to form robust interactions with the DNA substrate.

In total Ame-Lig makes direct contacts to 14 nucleotides in the nicked duplex (Figure 2B and C). As with other DNA ligases, the AD domain binds across the broken DNA strands with the nick positioned over the AMP-binding pocket in the center (Figure 2C). R50 bridges both strands, contacting the 5′PO_4_ and the backbone phosphate of the 3′OH of the nick. The 3′OH strand runs from the outer edge of the domain along a positively-charged groove created by the β3–α2 loop and the α3 helix. A second ionic interaction to the phosphate of nt31 is made by the R39 side-chain, while one position upstream, the phosphate of nt30 makes two contacts to N51, both from the side-chain and main-chain nitrogens, as well as to Oγ1. The hydrogen bond between N53 Nδ and the phosphate oxygen of nt29, three nucleotides upstream of the nick, marks the outermost contact with the 3′OH strand of the nick. The nt30 sugar makes a Van der Waals contacts from L98. The nt30 sugar also makes a Van der Waals contact with Leu98. AD-domain contacts to the 5′PO_4_ strand of the nick are limited to a hydrogen bond from Q16 in the β1 strand with the Nϵ2 forming a hydrogen bond with the vicinal phosphate of nt34, and an ionic interaction between R50 to both 5′phosphates on nt31 and nt32 (Figure 1E).The more extensive contacts between the AD-domain and the DNA-bound AMP that are essential for tethering the DNA in the active site are described below. The outer edges of the AD-domain form contacts to each end of the complementary strand including S86 and S90 from the α3- helix which inserts into the major groove between the complement and 3′OH DNA strands, as well as K15 and R203 from the the β1 strand the β7—the β8 loop respectively.

The complementary strand, runs the length of the OB domain in a 5′ to 3′ direction from the top, along a channel formed by side chains of K227, S236 and K248 on the major groove side, and K229, T251, S285, F283 and T276 in the minor groove (Figure 2B). The central span of the complement backbone is contacted by K227, K229, K248, G250, T276 and N278, and extensive interactions are made between the OB domain and five of the six complementary nucleotides opposite the nick, including hydrogen bonds from side-chains of T276 and N278 to nt14 and between the main-chain carbonyl of G250 the sugar moiety of nt11. A second basic pocket accommodates a two-nucleotide section of backbone from the 5′PO_4_ strand including an ionic interaction between the side chain of K271 and nt33, while the backbone of nt34 forms a hydrogen bond with the backbone nitrogen of E255 on the periphery of the domain. Specific side-chain contacts to the 5′ strand of the break include F283 positioned in the minor groove between the complement and 5′PO_4_ strand which stacks against the sugar of nt33, and S285 which forms a hydrogen bond to the ribose sugar of nt34 two- and three- nucleotides downstream of the nick respectively (Figure 2D). The margins of contact between Ame-Lig and the complementary strand are seven nucleotides up- and five nucleotides downstream of the nick position and are formed between side-chains of K15 and R203 to the phosphate of nt5, and side-chains of S86 and S90 which hydrogen bond with the phosphate and the sugar respectively of nt16.

### Nick binding interactions and conformations are conserved

**Figure 3.**
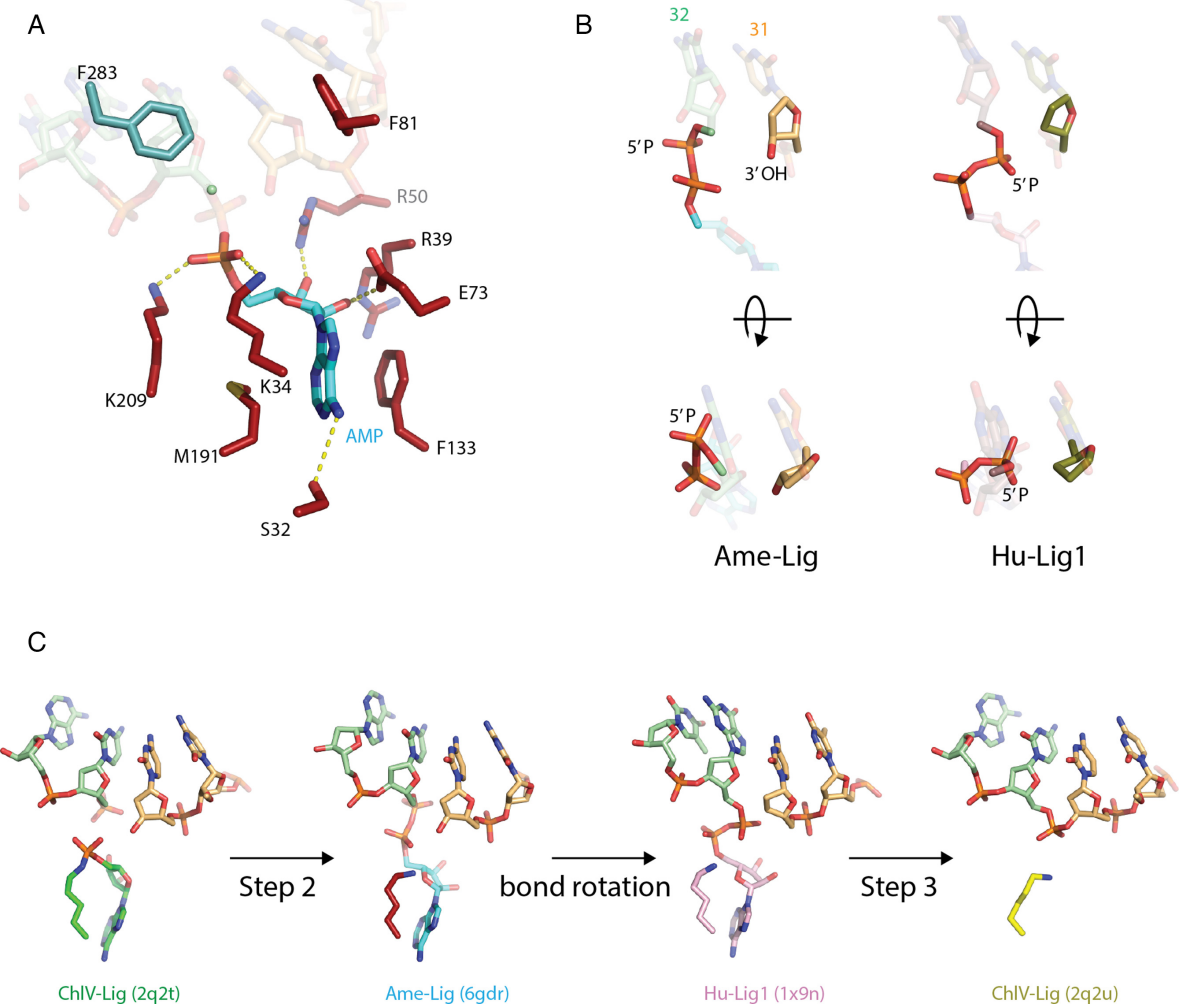
Active site of Ame-Lig. (**A**) side-chain interactions with the adenylated nick. AMP is shown in blue, AD-domain side-chain in red, OB-side chains in teal. Hydrogen bonds are indicated by dashed yellow lines. (**B**) Two views of the phosphodiester bond between the 5′ phosphate of the nick and the AMP highlighting the difference in orientation between the Ame-Lig structure (3′ terminus colored light orange) and Hu-Lig1 (3′ terminus coloured olive). Note that the latter structure was crystalized with a 3′ deoxy nucleotide at the nick terminus to prevent nick joining. (**C**) Steps of nick-sealing captured in different ligase–DNA complexes by X-ray crystallography. The DNA-bound enzyme adenylate of ChlV-Lig (4), the step 2 intermediates from Ame-Lig and Hu-Lig1 (8) before and efter phosphodiester bond rotation, and ChlV-Lig after step-3 nick sealing before product release.

Extensive contacts between the DNA-bound AMP moiety are essential for positioning the 5′PO_4_ strand for nucleophilic attack, and for tethering the complex in the active site. These include hydrogen bonds from the side chain of motif V residue K209 of to the alpha phosphate, motif III residue E73 to the 2′OH group of the ribose sugar, and the conserved R50 to the 3′OH (Figure 3). The rings of the adenine base are sandwiched between M191 of motif IV and F133 of motif IIIa. Motif I provides three further polar contacts from the sidechains of S32 to the exocyclic N6, K34 of the adenine ring and AMP phosphate and R39 to the ribose 2′OH.

The two terminal base pairs of the nick are distorted into an RNA-like A structure with the sugar moiety adopting a C3′ endo conformation and shortened distances between backbone phosphates, while the remainder of the duplex adopts the preferred DNA-B form. As is the case with other previously-described ligase–DNA intermediates, this distortion is a consequence of two conserved phenylalanines F81 and F283 stacking against the sugars of the 5′PO_4_ and 3′OH nucleotides. The characteristic DNA bending observed in other ligase structures is also preserved here, with the DNA portions of the Ame-Lig and ChlV-Lig structures (both nicked and sealed) being remarkably superimposable (Supplementary figures S5 and S6). This bending is less pronounced when compared with larger Human ligase 1 (Hu-Lig1) and Human Ligase 3 (Hu-Lig3) structures, presumably due to participation of their DNA-binding domains.

Examination of the diphosphate bond linking the AMP to the 5′PO_4_ indicates we have captured the step 2 intermediate immediately after transfer of the AMP from K34 to the 5′PO_4_ of nt32 and before reorientation of the AMP-nucleotide phosphodiester bond. In the Ame-Lig structure the 5′PO_4_ is orientated away from the adjacent terminus at a distance of more than 5Å from the 3′OH of nt31. For nucleophilic attack to occur, the activated phosphate must reorient almost 90° about the diphosphate-bond axis as seen in the app-DNA intermediate captured in the Human ligase1 (Hu-Lig1) structure, bringing it within 3 Å of the 3′OH (Figure 3B). In this way the AMP-Lig structure describes an additional conformation on the reaction trajectory towards formation of the new phosphodiester backbone bond (Figure 3C). Participation of the conserved arginine R39 (R42 in ChlV-Lig and R589 in Hu-Lig1) may be important in this transition as its coordination changes from the phosphate backbone of the 3′terminal nucleotide in the pre-Step-2 ChlV-Lig structure (2q2t) to the 3′OH of the AMP in the post-Step-2 Ame-Lig form, and then to the bridging oxygen of the DNA-AMP phosphodiester in the Hu-Lig1 structure, presumably stabilizing this rotated conformation. The linear product-bound configuration of ChlV-Lig (2q2u) shows the sidechain has returned to the orientation observed in the DNA-bound enzyme adenylate form. No metal ions were resolved in the present structure, which is consistent with addition of EDTA during crystallization conditions to prevent step 3 catalysis. Attempts to determine metal binding sites or later reaction intermediates by soaking with MgCl_2_ prior to cryoprotection were unsuccessful, giving rise to poorly diffracting crystals.

### Unique DNA binding interactions form conserved motifs in Lig E

The majority of protein-DNA contacts are conserved between the minimal Ame-Lig, the small latch-bearing ChlV-Lig and the large Hu-Lig1 and Hu-Lig3 enzymes with their helical DNA-binding domains. However, additional binding interactions identified in the Ame-Lig structure appear to be highly conserved among the Lig E-type ATP-dependent DNA ligases and may account for their ability to bind DNA without a latch or DNA-binding domain. These include the lysine-bearing motif at the apex of the OB domain between the β9 and β10 strands, which is replaced by the 29-residue latch in ChlV-Lig. As seen in the previous Psy-Lig enzyme-adenylate structure, this short inter-strand segment of Ame-Lig forms a 3_10_ helix positioning the lysine pair in a fork straddling the complement strand with the side-chain of K227 positioned alongside the complement backbone and that of K229 pointing into the minor groove between the complement and the 5′PO_4_ strand. The latter residue forms two hydrogen bonds to nt9 from the side-chain amine to the ribose O4′, and from the main-chain amide to the backbone phosphate as well as extensive Van der Waals interactions (Figure 2B). A long-range polar contact to the base of nt35 with a length of 4.7 Å is also possible, making this the only residue to interact with both strands of the duplex. The short-range contacts of K227 are less extensive, limited to a main-chain interaction with the phosphate of nt11, which is complementary to the 5′position of the nick, and Van der Waals interactions; however long-range polar contacts (5.0 Å) are possible from the side-chain amide to both backbone phosphates of nt12. Both K227 and K229 have main-chain amide interactions with the phosphate backbone, from K227 to the nt11, and from 229 to nt10.

A second key region of interactions on the OB domain are the polar side chains of K248 T251 and E255 which point into the minor groove, while this β11–α7 loop is stabilized by a series of hydrophobic interactions between F247, L249 and F253 on the reverse side. K248 has main-chain interactions with the phosphate of nt13, the sugar of nt12, and its side-chain has a long-range ionic interaction with the phosphate of nt12 (5.0 Å) and nt11 (5.0 Å). Nucleotide11 and nt12 are complementary to the 5′ and 3′ ends of the nick respectively. The side-chain hydroxyl of T251, is in hydrogen bonding distance of both O4′of nt34, and the base of nt33, one position upstream of the nick. L89 which makes Van der Waals contacts with the ribose sugar of nt30 adjacent to the 3′ end of the nick occupies an equivalent position to R80 of Psy-Lig in the AD domain.

A multiple alignment of more than 500 Lig-E sequences reveals that these two motifs G-K-G-K-Aromatic and F-Basic-I-G-S-G-F-x-D are highly conserved (Figure 2F). Structural alignment indicates a lower degree of conservation in other ligases, for example the first motif containing a lysine fork is not found in ChlV-Lig, the Human ligases 3 and 4 (Hi-Lig3 and Hu-Lig-4) or ligases from *Sulfolobus solfataricus* and T7, while the second larger pattern is absent in Hu-Lig3, Hu-Lig 4 and T7 structures.

### Structure-guided mutagenesis reveals essential binding interactions

The importance of specific side-chain to DNA interactions of Lig E activity was further investigated for a sub-set of positions by mutating single residues to alanine in the close homolog Psy-Lig. The choice of positions to be mutated was based on a combination of modelled DNA-binding predictions from the enzyme-adenylate structure of Psy-Lig in our previous work (12), and on analyses of the Ame-Lig-DNA complex. It includes the two lysines in the small loop motif that replaces the ChlV latch, K191 and K193 (Ame-Lig K227 and K229), as well as K212 and S215 (Ame-Lig K248 and T215) in in β11 of the OB domain. A single position R80 (Ame-Lig L89) in the AD-domain was also investigated for its DNA-binding contribution, as well as K25A, the site of covalent adenylation.

**Figure 4.**
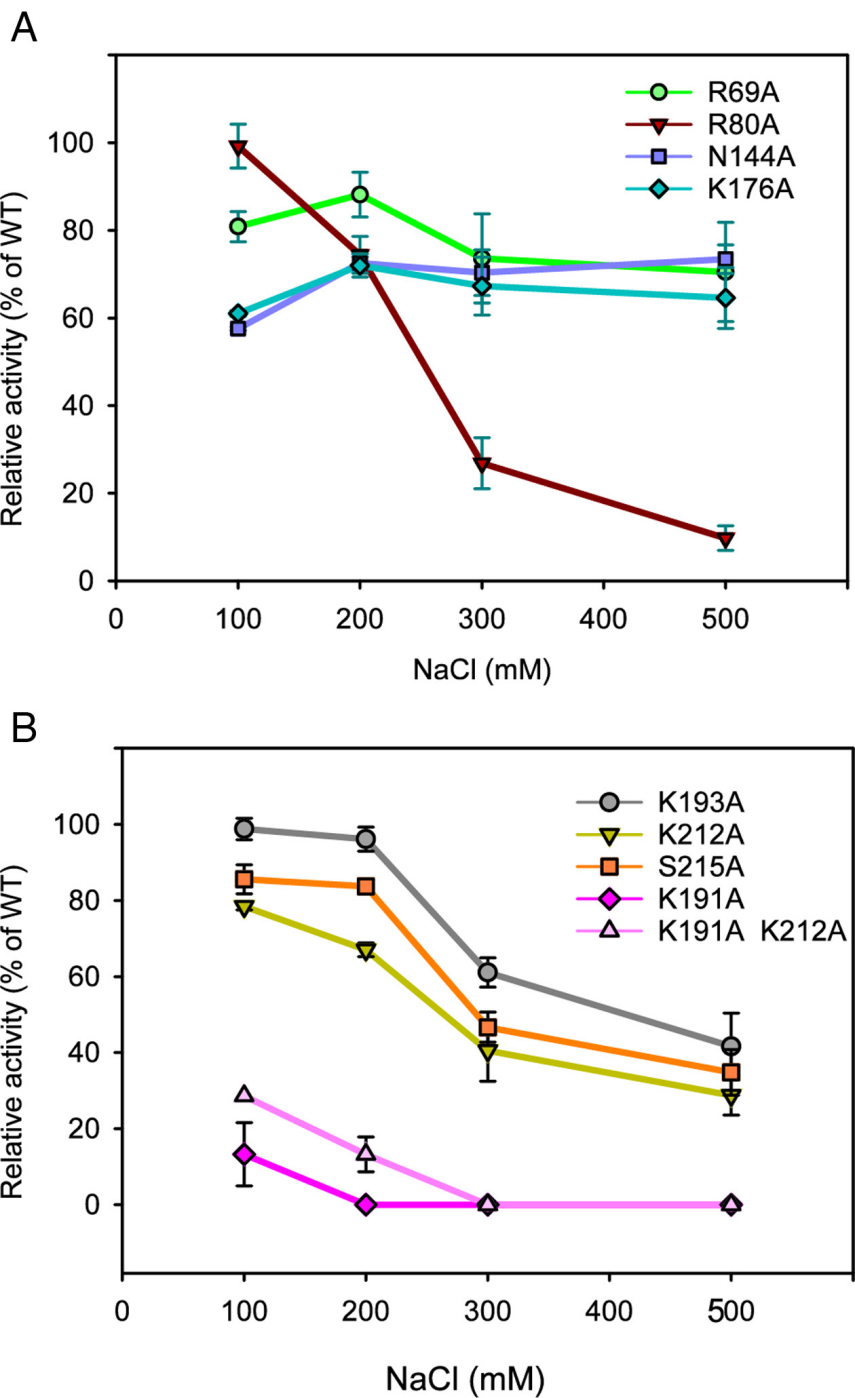
NaCl dependence of Psy-Lig point mutants. Ligase activity is measured by MB assay, each data point is normalized to WT activity at the same NaCl concentration. Measurements are the mean of three replicate experiments; error bars represent the standard deviation from the mean. Raw data, including the NaCl dependence of the WT enzyme are given in Supplementary Figure S3E.

**Figure 5.**
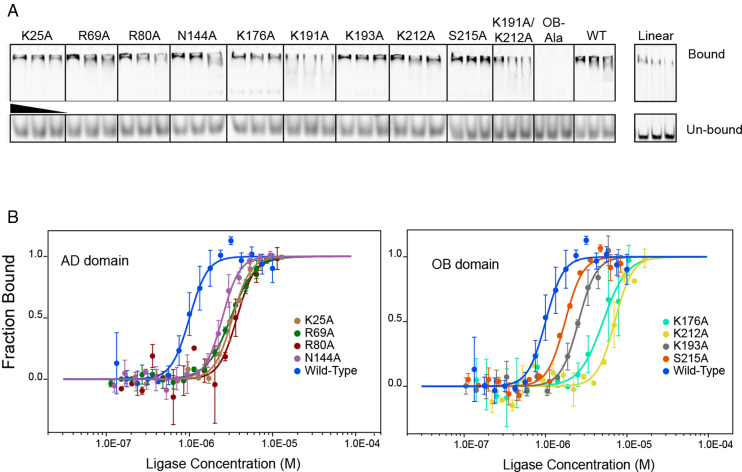
Binding affinity of Psy-Lig mutants for nicked DNA. (**A**) EMSA with FAM-labeled DNA incubated with a 40×, 20× or 10× molar excess of DNA ligase. Bound DNA is shown in the upper panel, free DNA from the same lane in the lower. WT Psy-Lig binding to linear DNA is shown in an adjacent panel. (**B**) MST binding curves of Psy-Lig AD-domain mutants (left) and OB-domain mutants (right).

These mutations decreased the specific ligase activity of Psy-Lig by up to 50%, and in the case of K191A by more than 80%. K25A was, as expected, catalytically inactive. Increased NaCl concentration has a marked effect on ligase activity of mutations to DNA-binding residues R80, K191, K193, K212 and S215 relative to the WT. This indicates that these residues contribute significantly to the collective electrostatic interaction between protein and DNA as removal of single positions renders the remaining interactions more sensitive to charge screening by salt ions (Figure 4). The Psy-Lig K191A/K212A double-mutant was equally defective in ligation to the single K191A mutant, however a variant where all four positions on the OB-domain were mutated (K191/K193/K212/S215, hereafter OB-Ala) was completely inactive. The effect of these mutations on DNA binding further indicates a key role for these residues, with EMSA confirming that binding is significantly reduced in both the single (K191A) and double (K191A/K212A) mutants and entirely abolished for the OB-Ala variant (Figure 5A). K25A was able to bind DNA (Figure 5A), although with decreased affinity (Figure 5A and B). EMSA experiments with un-nicked DNA indicate that wild-type Psy-Lig binds the linear product, but very poorly.

DNA-binding affinities quantified by MST show that all mutations had a negative impact on binding affinity, with increases of between two- and 7-fold for EC50 values (Figure 5B, Table 3). The biggest effect on measurable affinity was seen for the single K212A mutant, while no binding was measurable by MST for any single (K191A), double (K191A/K212A) or quadruple (OB-Ala) variants of K191. Binding of wild-type Psy-Lig to linear DNA was also not detectable by MST.

**Table 3.** Binding affinity of Psy-Lig mutants to nicked DNA measured by MST. Values are the average of four replicates, error is given as standard deviation of the mean

Psy-Lig mutant	EC_50_ (μM)
WT	0.98 (± 0.042)
K25A	3.30 (± 0.110)
R69A	3.08 (± 0.142)
R80A	3.62 (± 0.34)
N144A	2.45 (± 0.10)
K176A	5.17 (± 1.55)
K191A	ND*
K193A	2.55 (± 0.110)
K212A	7.10 (± 0.076)
S215A	1.74 (± 0.087)
K191A/K212A	ND*
OB-Ala	ND*

*ND = not determined.

### Linker region plays a role in DNA binding

**Figure 6.**
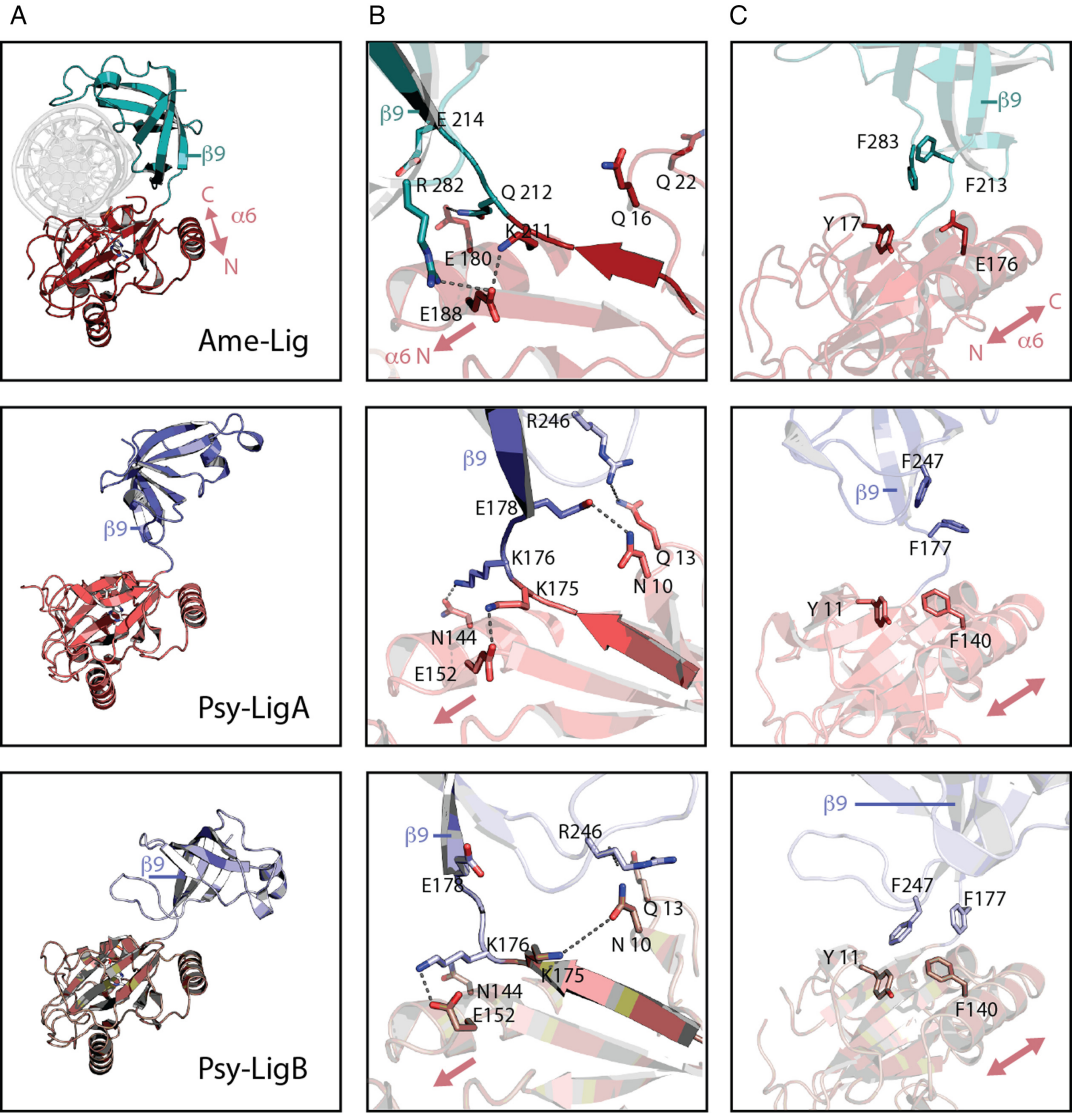
Comparison of Lig E domains in DNA-bound and apo- conformations. AD-domain is shown in red, OB domain is shown in cyan/blue. Polar contacts are indicted by dashed gray lines. (**A**) Overall domain conformations. (**B**) Rearrangement of hydrogen bonding patterns between three conformations. (**C**) Comparison of hydrophobic interactions between three conformations.

In our previous report on the enzyme-adenylate structure of Psy-Lig, two conformers denoted Psy-LigA (partially open) and Psy-LigB (fully-open) were observed in the asymmetric unit, both in the ‘open’ conformation with the OB-domain deflected away from the adenylation site on the AD-domain (12). With the closed DNA-bound structure of Ame-Lig we can describe a third conformation in which the AD- and OB-domains are swivelled to wrap about the DNA (Figure 6A). This open-to-closed transition has been described previously for the enzyme-adenylate and DNA-bound forms of ChlV-Lig and is considered to be essential for ensuring that the active site in its adenylated form is exposed and available to receive nicked DNA (24). In Psy-Lig, residues in the linker form different hydrogen bonding patterns between the two open A- and B-conformations. These residues are conserved or equivalently substituted in Ame-Lig, and are involved in a different set of interactions in the closed structure.

The most open structure is Psy-LigB where the OB domain is tilted furthest from the AD active site. Here, K175 and K176 of the linker form interactions with the AD-domain through a hydrogen bond with N10 and a salt bridge with E152 respectively. In the partially open conformation Psy-LigA, these interactions are rearranged by a one-residue turn about the linker giving rise to a new interaction between K176 and N144 of the AD-domain, while K175 interacts instead with E152. Its former bonding partner N10 forms a hydrogen bond with D178 of the linker, directly proximal to the OB domain and an inter-domain interaction is formed between R246 of the OB domain and Q13 of the AD-domain. In the DNA-bound Ame-Lig structure the two interactions between the linker and the AD-domain equivalent to those observed in Psy-LigA are retained; K212-E218 (Psy-Lig K175-N152) and E180-N212 (Psy-Lig N144-K176). The linker-AD domain interaction between Q16 and E214 (Psy-Lig N10-E178) and the inter-domain bond between Q22 and R282 (Psy-Lig Q13-R246) are lost upon domain reorientation to the closed form. R282 (Psy-Lig E152) instead forms a new interdomain bond with E188 (Figure 6B) while Q16 hydrogen bonds to the phosphodiester backbone of the DNA substrate as described above. Q16 and Q22 are now solvent exposed and our inability to resolve two intervening residues S21 and R22 in the Ame-Lig structure likely reflects the flexibility of this loop.

In addition to these polar interactions, the domain orientations of Psy-LigB, the most-open configuration, are stabilized by inter-domain pi-pi stacking from a trio of phenylalanines F140 to F177 and F247. The latter has an additional inter-domain Van der Waals contact, with Y11 of the AD-domain. In Ame-Lig an acidic residue E176 is found in place of the central AD-domain phenylalanine (F140 in Psy-Lig), therefore no stacking interaction is possible between Ame-Lig Y17 and F123 (Psy-Lig Y11 and F177). However, Ame-Lig F283, which has Van der Waals contacts with DNA in the bound form, and Y17 would form equivalent interaction to the Psy-Lig Y11-F247 pair when Ame-Lig assumes a fully-open conformation (Figure 6C). These aromatic interactions are not present in either the less-open Psy-LigA conformation or the DNA-bound closed Ame-Lig conformation.

In summary, all three conformations represented by Psy-LigB, Psy-LigA and Ame-Lig appear to be stabilized by non-covalent interdomain interactions; two hydrogen bonds and at least one aromatic interaction in the most open (Psy-LigB) state, four hydrogen bonds in the less-open (Psy-LigA) state, and two hydrogen bonds, in addition to DNA-mediated interactions, in the closed (Ame-Lig) form.

To investigate the role of inter-domain interactions in facilitating DNA binding, individual mutations were made to Psy-Lig, N144 and K176 (Ame-Lig E180 and Q212); the two residues observed to form hydrogen bonds between the α6 helix of the AD-domain and the inter-domain linker in the enzyme-adenylate structure. The conserved Psy-Lig R69 (Ame-Lig, R78) of the β4-α3 loop of the AD-domain was also mutated as it forms hydrogen bonds with both the α6 helix through the main-chain oxygen of G149 (Ame-Lig S185), and to E24 (Ame-Lig E33) of motif I, directly adjacent to the catalytic lysine residue, thus it has potential to coordinate interactions between these key regions. Mutation at either position of the Psy-Lig linker-AD hydrogen bond resulted in a >40% decrease in ligation, while the R69A mutant retained 80% of WT activity (Figure 4). Moderate decreases in DNA-binding affinity were detected by MST for all three mutants (Figure 5B), however no NaCl sensitivity relative to the WT Psy-Lig was observed, consistent with these mutants disrupting the protein structure, rather than binding electrostatics with DNA (Figure 4). The more severe effect of mutating the K176 member of the pair than N144 may be rationalized as K176 participates in H-bonds in both the Psy-LigA (partially open) and Psy-LigB (fully-open) forms, suggesting that the both conformations may play a role in DNA engagement. Furthermore, the impact of removing the side-chain of Psy-Lig R69 which forms an intra-domain interaction between α-6 and the adenylation motif suggests this is important for maintaining the correct protein conformation for DNA binding.

### Mutant stability depends on cofactor and metal ion concentration

**Figure 7.**
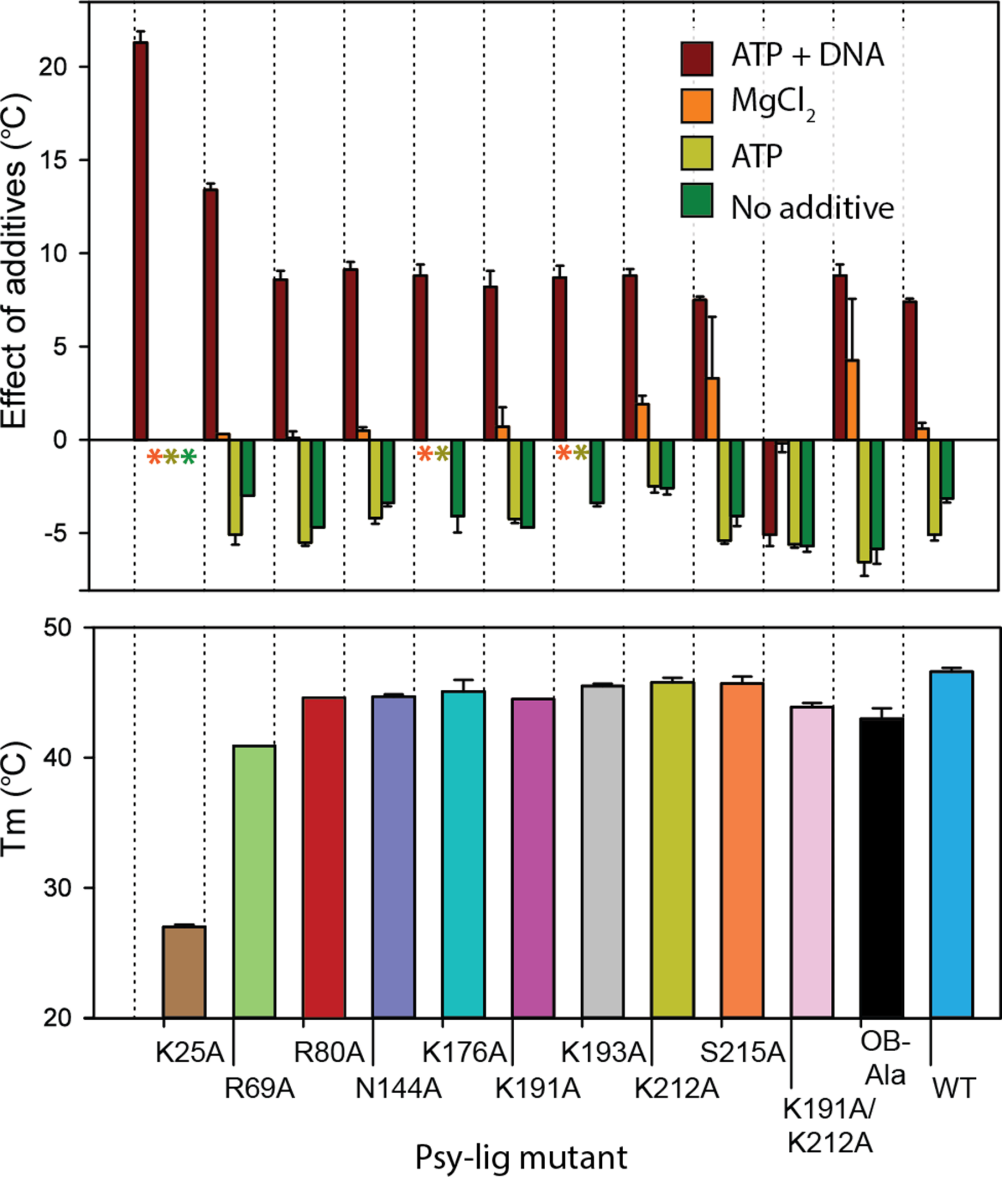
Stability of Psy-Lig mutants measured by DSF. In the case that multiple transitions were observed, *T*_m_ values are given for the higher temperature, with the exception of K25A which had a single low *T*_m_. (**A**) Difference in *T*_m_ with additives; reference condition is 0.1 mM ATP, 10 mM MgCl_2_. Asterisks mark conditions where no high-temperature transition was observed. (**B**) *T*_m_ of mutants in buffer C (50 mM Tris pH 8.0, 100 mM NaCl, 1 mM DTT, 5% glycerol) without any additives.

Correct folding of all Psy-Lig mutants was confirmed by CD spectroscopy (Supplementary Figure S3B). Under standard conditions measured by DSF, both mutants and WT exhibited a major unfolding event between 40 and 45°C, with the exception of the K25A catalytic mutant which unfolded below 30°C (Figure 7). NaCl concentration had no impact on thermal stability indicating that the decrease in activity of DNA binding-site mutants was not due to destabilization of the protein under high salinity conditions (data not shown). DSF did however indicate mutant-specific differences in stability depending on the presence of cofactors or substrates. Addition of ATP and MgCl_2_ increased stability of the WT and most mutants by approximately 5°C (Figure 7). Addition of MgCl_2_ alone was stabilizing for K191A, K212A and OB-Ala, but ATP alone was destabilizing in all cases. Interestingly, for several mutants (N144A, K176A, K193A and K212A) omission of any additives, or addition of ATP or MgCl_2_ separately lead to the appearance of an additional lower temperature transition (Supplementary Figure S3C). All mutants except the double mutant K191A/K212A exhibited a significant increase in stability in the presence of DNA/ATP, which in the case of K25A was >20°C. Unlike the other mutants, K25A consistently lacked an unfolding transition >30°C, except in the presence of DNA, suggesting that the thermal stability of Psy-Lig is influenced by adenylation state.

To test whether impaired adenylation could account for the loss of activity, Psy-Lig mutants were assayed at increased ATP concentrations. However, addition of a 5-fold excess of ATP under otherwise standard reaction conditions did not lead to noticeable increases in activity or rescue poorly-active variants, indicating that neither mutations targeting DNA-binding residues or linker interactions decreased affinity for ATP (Supplementary Figure S3 D).

## DISCUSSION

Here, we unequivocally demonstrate that Lig E binds DNA stably without participation of appending DNA-binding domains or ordering of unstructured loops. The DNA-binding footprint of Ame-Lig, (8 nucleotides and 12 nucleotides on the nicked and complement strands respectively) is two nucleotides fewer on the 3′OH strand than the previously-characterized minimal ATP-dependent DNA ligase from *Chlorella* virus (ChlV-Lig) and lacks additional contacts within these boundaries; in particular the three nucleotide stretch on the open duplex face of the complement. In the ChlV-Lig structure, contacts to these positions are supplied by residues within the latch module, and by the DNA-binding-domains of larger ligases such as Hu-lig1, Hu-lig3 or the NAD+-dependent housekeeping bacterial ligases (4,8,25,26).

The present the crystal structure of Ame-Lig bound to adenylated DNA combined with structure-guided mutagenesis study of Psy-Lig confirms that the Lig E class of ATP-dependent DNA ligases binds DNA without complete encirclement of the duplex using a combination of specific charge-pair interactions on the well-structured globular OB domain of the protein. Several of these interactions are unique with respect to previously-described ligases and highly conserved among other Lig Es. In particular, the G-K-G-K-F motif that replaces the latch region of ChlV-Lig (Figure 2D) and contains interacting lysines Ame-Lig K227 and K229 (Psy-Lig K191 and K193), and the F-Basic-I-G-S-G-F-x-D position on the OB domain near to the linker which contains Ame-Lig K248 and T251(Psy-Lig K212 and S215; Figure 2E). Mutagenesis studies demonstrate that these charged residues on the OB-domain are important for Lig E- DNA interaction, and mutation of all four in parallel completely abolished DNA binding. A clear point is that mutation of residues interacting with complement-strand nucleotides that are base-paired to the ends of the nick are significantly more deleterious than those at more distal sites. Specifically, mutations to Psy-Lig K191 (Ame-Lig K229) and Psy-Lig K212 (Ame-Lig K248) are the most severe individual mutations tested here, although they are not additive. The importance of the long-range polar side chain interactions of Ame-Lig K229 (Psy-Lig K191) with DNA are underscored as mutation at this position caused an 80% decrease in activity, complete loss of salt tolerance and extremely defective binding (below the detection limit of MST). These interactions involve bridging phosphates to nucleotides complementary to both the 5′ and 3′ positions of the nick. The side chain amide of Ame-Lig K248 (Psy-Lig K212) is the only short-range polar contact to the complementary nucleotide base-paired to the 3′ position of the nick, with binding further strengthened by side-chain Van der Waals interactions. Mutation of Psy-Lig K212A gave the most significant effect on binding affinity that could be quantified MST and caused loss of salt tolerance. Contribution of other interactions can be rationalized from the Ame-Lig structure; Ame-Lig K229 (Psy-Lig K193) has polar interactions with both the complementary and 5′ strands via a hydrogen bond to sugar and long-range polar interaction respectively. Ame-Lig T251 (Psy-Lig S215) makes the only contact with the base moiety of the DNA through its side-chain, an interaction that is conserved in its serine counterpart in the DNA-bound ChlV-Lig structure and has additional interactions with an adjacent ribose sugar.

In addition to illuminating the mode of DNA interaction by minimal Lig E ligases, the Ame-Lig structure has captured a second conformation of the Step-2 intermediate immediately after DNA adenylation, prior to re-orientation of the DNA-AMP phosphodiester bond for Step-3 catalysis. This demonstrates inversion of the phosphate centre of the AMP, but suggests that shortening of the inter-phosphate distances prior to Step-2 occurs through movement of the catalytic lysine-AMP phosphoamide bond toward the 5′PO_4_ rather than movement of the 5′PO_4_ as was previously proposed (4). Unanswered questions remain about the final Step-3 catalysis, which would be partially resolved by the capture of states immediately prior to the post-Step-3 ChlV-Lig structure (2q2u) where the enzyme remains engaged with the linear product but the ‘spent’ AMP cofactor has diffused from the active site. In particular a structure with the cofactor retained in the binding pocket, or where added metal ions can be resolved would be highly informative, especially if combined with more extensive mutagenesis studies to, for example elucidate the role of the conserved R39 in 5′PO_4_ orientation and AMP leaving.

The most salient difference between the Lig E DNA-bound structure and the minimal viral ChlV-Lig is there is no ordering of unstructured regions for Lig E upon interaction with DNA and no circumferential encirclement. Although Ame-Lig has a probable loop region, this was not observed to participate in DNA binding from the crystal structure, and deletion or mutation of this segment had no impact on steady-state ligase activity. The lack of disordered-to-order transitions or other local structural changes is underscored by the high structural identity between Ame-Lig and Psy-Lig domains when these are aligned individually, suggesting that the DNA-interaction surface of the enzyme-adenylate is pre-organized for substrate binding. As a consequence, the most significant region of plasticity in Lig Es during the binding process is the linker region.

Changes in the hydrogen-bonding pattern of the linker region are implicated in coordinating rearrangement of the domains relative to each other to form the C-shaped clamp observed in the Ame-Lig structure. Residues of the linker region which comprises nucleotidyl transferase motif V are already known to be important for activity: the first conserved lysine of motif V (K173 in Ame-Lig, K209 in Psy-Lig) forms hydrogen bonds with the a-phosphate of the AMP in the enzyme-adenylate, and both conserved lysines (Psy-Lig K173/K175 and Ame-Lig 209/K211) stabilize the step 2 intermediate by coordinating non-bridging oxygens on the AMP phosphate in the DNA-bound structure (4). The present study highlights an additional role of stabilizing contacts between the linker and the domains including hydrogen bonds and salt bridges, as well as polar interactions. It is notable that although the individual residues involved in linker-domain bonds are not strictly conserved among homologs, the potential for charge-pair or hydrophobic interactions is generally preserved, providing further support for a functional role of these interactions (Supplementary Table S3). The significant effect of mutating Psy-Lig K176A which features in interactions of both the B (most open) and A (partially-open) forms of enzyme-adenylate suggests that both conformers play a role in activity. Superposition of the AD-domain of the Psy-Lig B conformation with DNA-bound structure of Ame-Lig form places a 12 residue loop of Psy-Lig in the major groove of the DNA substrate where polar residues are positioned to interact with backbone phosphates (Supplementary Figure S6). It is possible that these interactions help position the DNA at the enzyme-adenylate binding interface, and the rearrangement of hydrogen bonds described here acts as a conformational switch between the open and closed states, reorienting the domains. Such additional interactions may be necessary to ensure tight binding in the absence of an intrinsic nick-sensing mechanism such as the latch of ChlV-Ligase or human ligases.

Surprisingly the K25A mutant was able to bind to DNA, albeit with decreased affinity. This is in contrast with ChlV-Lig, which requires adenylation at the active site for nick sensing, but has been reported for other viral ligases such as T4 where an analogous lysine to leucine substitution at the catalytic site could bind nicked DNA, and T7 DNA-ligase where a truncated protein that eliminated the catalytic lysine was still able to bind DNA (7,24,27,28). The T7 DNA-ligase is especially interesting in this respect, as like the bacterial Lig E-type ligases, it lacks an extensive DNA binding domain or long latch-like insert. The crystal structure of the apo-enzyme show it possesses two unstructured loops in the AD- and OB-domains, each 9 residues long, which may be involved in DNA binding but are situated between different secondary structure elements compared with the ChlV-Lig latch or the unstructured Ame-Lig loop (27). Incredibly, truncation studies show that the individual domains of T7 DNA ligase have intrinsic double-strand binding capacity suggesting and their specificity for high-affinity nick binding appears to be cooperative (29). This suggests that in DNA ligases where complete duplex encirclement is not a prerequisite for ligase–DNA engagement, intrinsic binding determinants residing in individual domains may impart these with independent DNA-binding abilities, and it would be interesting to investigate whether the same applies to the Lig E class of DNA ligases.

It is interesting to consider the implications of these findings for understanding the evolution of DNA ligases. In foundational papers describing minimal ATP-dependent DNA ligase interaction with DNA, the Shuman group propose that the larger cellular ligases evolved by domain fusion to minimal pluripotent enzymes such as ChlV-Lig, presumably with acquisition of large helical domains accompanying loss of nick-sensing latches and loops (4,9). Our previous phylogenetic analyses of Lig E proteins indicate these may have evolved from a T4 phage-like ancestor that possessed an N-terminal DNA-binding domain and that proteobacterial Lig Es represent a minimised form as a result of domain truncation (10). It would be extremely interesting to examine the structural basis of such bacteriophage ATP-dependent DNA ligases binding to their substrates; to date no protein structures exist for T4 phage-like ATP-dependent DNA ligases either with or without DNA.

In summary, the Lig E-type ATP-dependent DNA ligases, epitomised by the Ame-Lig and Psy-Lig proteins studied here, preserve all essential attributes of functional DNA ligation in a minimal scaffold. This includes robust substrate engagement, discrimination between nicked and linear DNA, bending of the nicked duplex, and distortion of the terminal nucleotides to the A-form.

## DATA AVAILABILITY

Atomic coordinates and structure factors for the reported crystal structures have been deposited with the Protein Data bank under accession number 6GDR.

## Supplementary Material

Supplementary Data
