# Supplementary material for: DNA binding with a minimal scaffold: structure–function analysis of Lig E DNA ligases
Source: Nucleic Acids Res. 2018 Jul 11;46(16):8616–29. doi: 10.1093/nar/gky622 (PMC6144786; doi:10.1093/nar/gky622)
Supplement: Supplementary Data [file gky622_supplemental_files.pdf]

Supplementary information

Sup. Figure 1

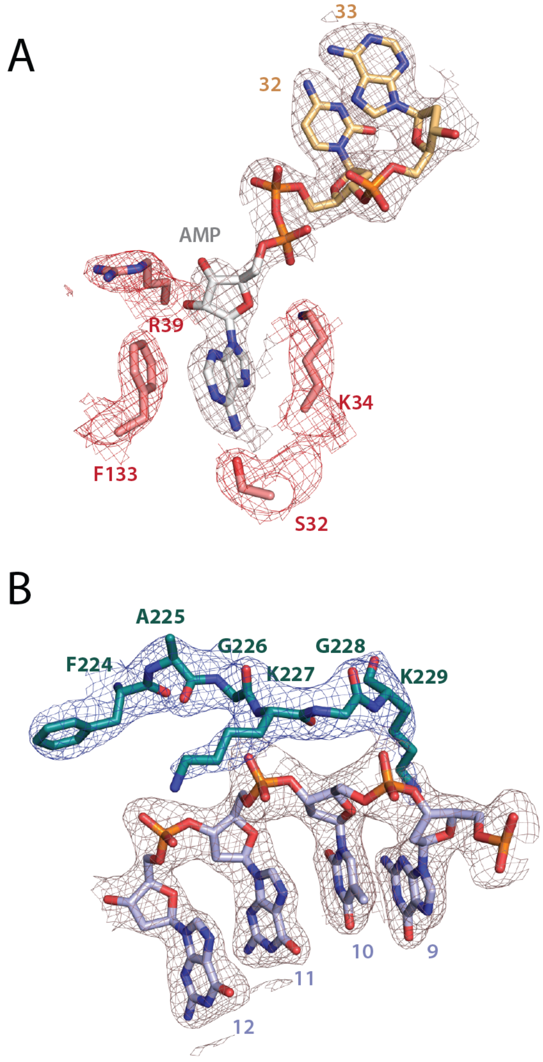

Supplementary figure 1. Observed electron density maps for Ame-Lig bound to DNA. 2Fo − Fc maps are displayed at 1.0σ. (A) Adenylated nicked DNA (grey mesh, gold/ white sticks) in the active site of Ame-Lig (red mesh, salmon sticks). (B) Interaction site of the OB-domain GKGK motif (blue mesh, teal sticks) with complement-strand DNA (grey mesh, purple sticks).

Sup. Figure 2

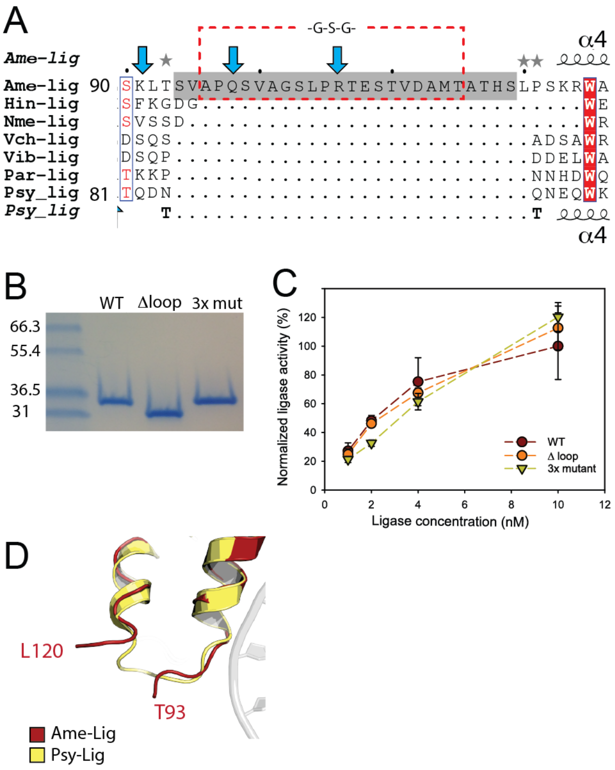

Supplementary figure 2. Non-conserved region of Ame-Lig. A) Alignment of a selection of Lig E-type ligases. The disordered region of Ame-Lig with no observable electron is shaded in grey. The region deleted in the Δloop mutant is boxed with a red dashed line, and the positions of residues converted to alanine in the 3x mut (K91, Q98, R106) mutant are indicated with arrows. B) SDS-PAGE of mutants confirming the deletion size of the Δloop variant. C) Activity of Ame-Lig variants measured by molecular beacon assay under standard conditions. D) Structural superposition of Ame-Lig and Psy-Lig highlighting the position of the unresolved section relative to secondary structure.

Sup. Figure 3

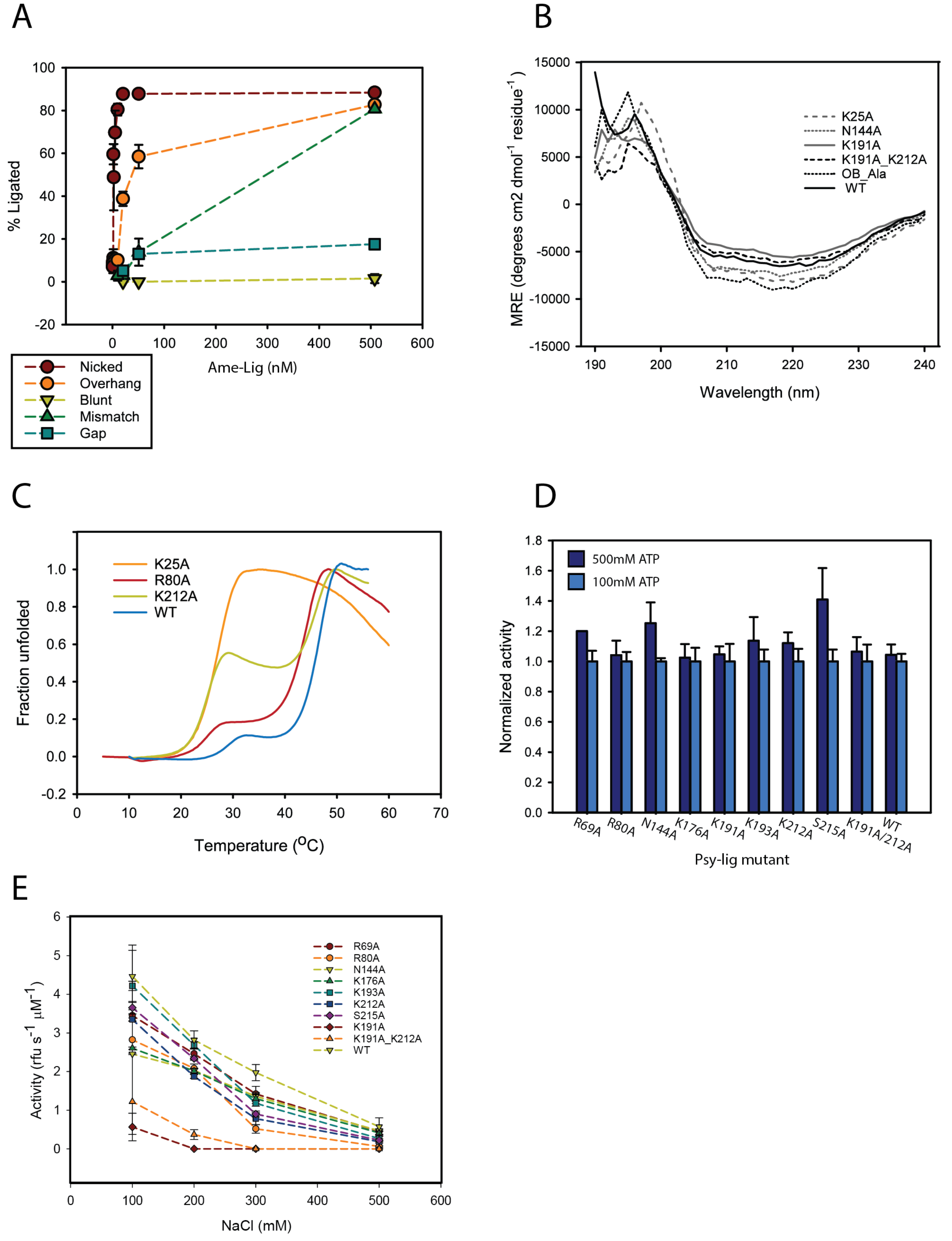

Supplementary figure 3. A) Ligase activity of Ame-Lig on various double-stranded substrates measured by denaturing urea-PAGE endpoint assay as described in (12). Nick and overhang substrates were tested for 15 minutes at 25 °C, other substrates were assayed at 15 °C for 2 hours (mismatch) or 6 hours (gapped and blunt). B) Representative CD spectra of Psy-Lig mutants. Data for remaining mutants is essentially identical. C) DSF unfolding data normalized to fraction folded for representative Psy-Lig mutants in the absence of added cofactor or substrate. D) Relative ligase activities of Psy-Lig mutants

measured by molecular beacon assay under standard (100 mM ATP) or high (500 mM) ATP conditions. E) Activities of Psy-Lig mutants with different concentrations NaCl measured by rea-time molecular beacon assay.

Sup. Figure 4

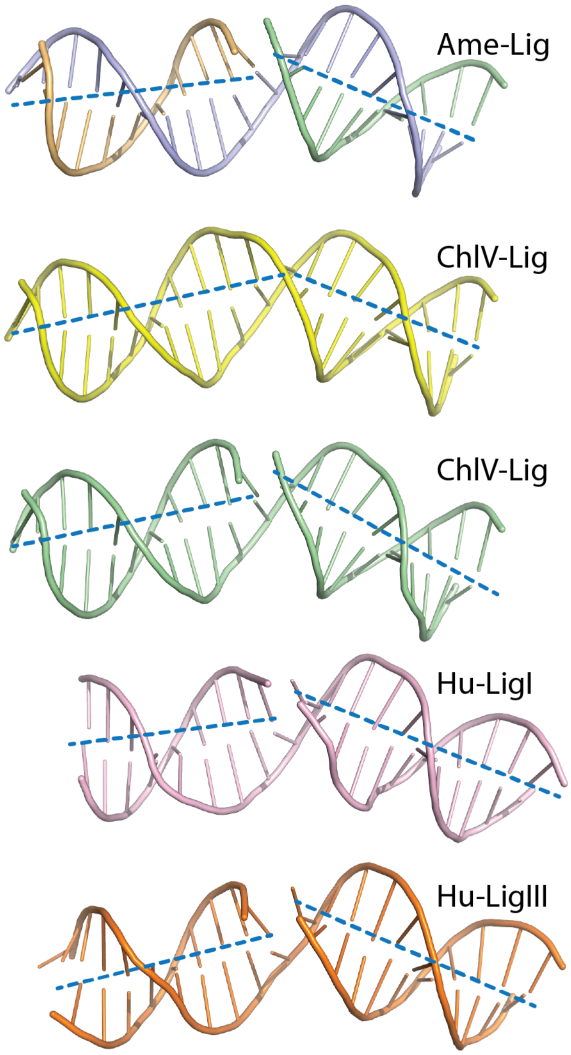

Supplementary figure 4. Cartoon representations of ligase-bound DNA showing the extent of bending. PDB files are as follows: Ame-Lig, 6gdr; ChlV-Lig linear, 2q2u; ChlV-Lig nicked 2q2t; Hu-Lig1, 3l2p; Hu-lig3, 1x9n.

Sup. Figure 6

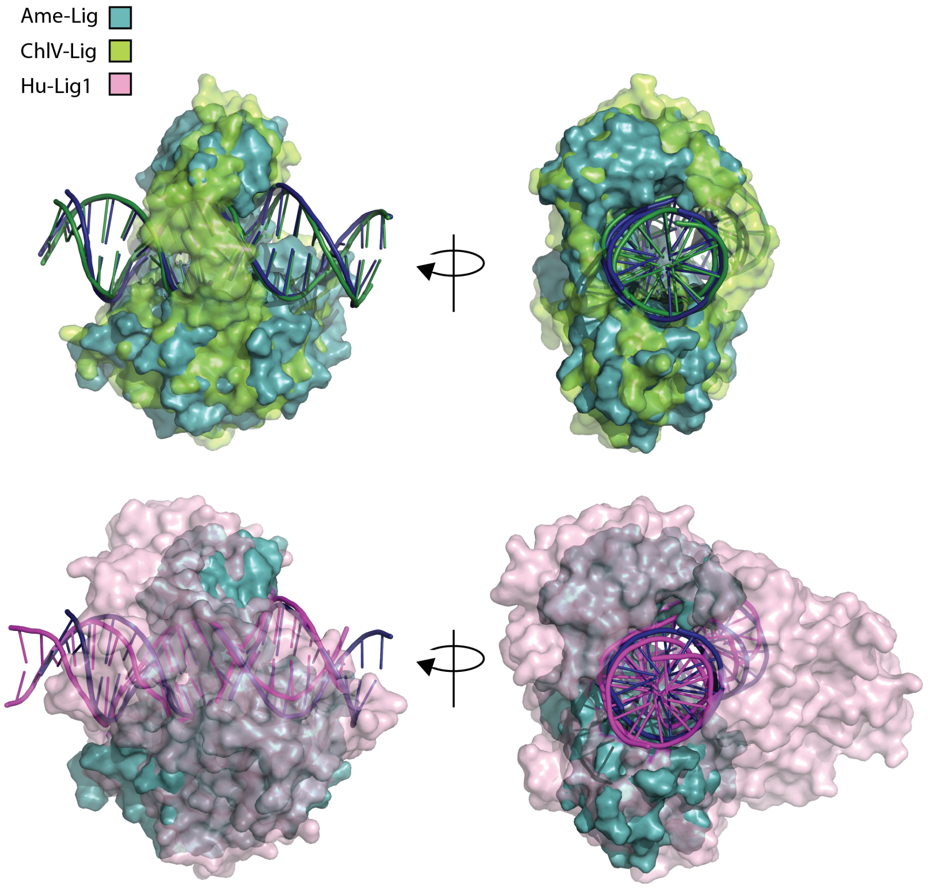

Supplementary figure 5. Superimposition of Ame-Lig with the larger ChlV-Lig (upper) and Hu-Lig1 (lower) highlighting the complete encirclement of DNA by the latter structures. DNA is coloured dark blue for Ame-Lig, green for ChlV-Lig and dark pink for Hu-Lig1.

Sup. Figure 5

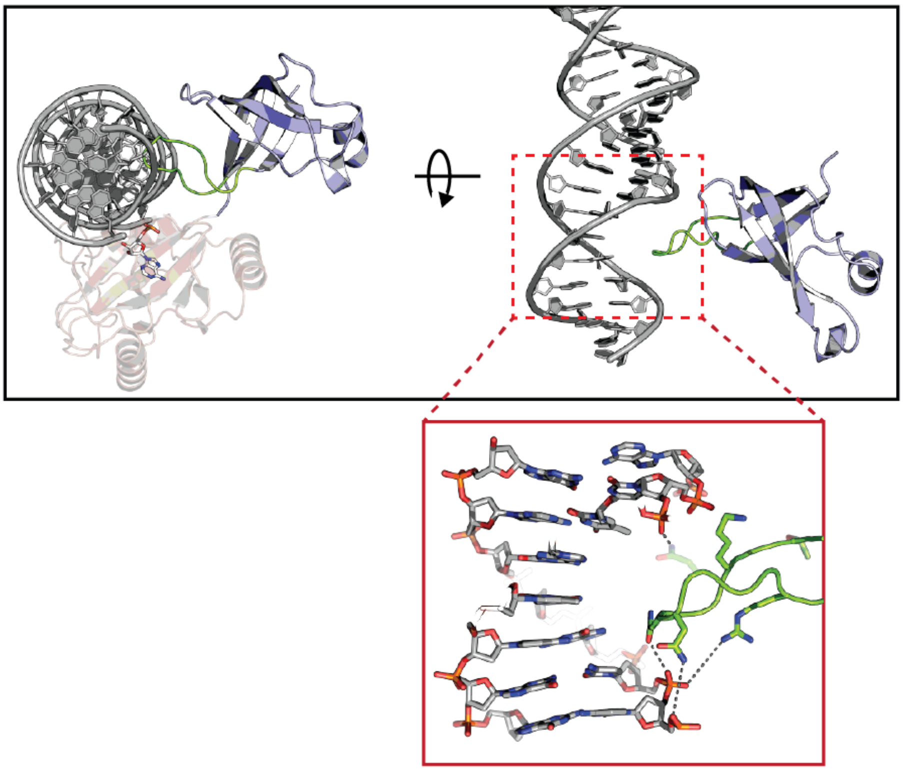

Supplementary figure 6. Superposition of the B conformation of the Psy-Lig enzyme-adenylate with the DNA-bound form of Ame-Lig by alignment of the ligase AD-domains. This places a loop on the back of the Psy-Lig OB domain (green) in the major groove of the DNA.

Sup. Table 1

Supplementary Table 1. Oligonucleotides used in experimental work. All were purchased from Sigma as reversed HPLC quality.

Purpose	Name	Sequence	Modification
Psy-lig R69A	R69A_FD	atggcgagttatggattgctgctcaacagttcgaaaccg	
	R69A_BK	cggtttcgaactgttgagcagcaatccataactcgccat	
Psy-lig K191A	K191A_BK	cacctaacagaccttcatatttgcctgcacccggcaaatagg	
	K191A_FD	cctatttgccgggtgcaggcaaatatgaaggtctgttaggtg	
Psy-lig K193A	K193A_BK	cacctaacagaccttcatatgcgcctttacccggcaaatagg	
	K193A_FD	cctatttgccgggtaaaggcgcatatgaaggtctgttaggtg	
Psy-lig K212A	K212A_BK	gagaatccagagccgattgcgaaggttacgccttcctc	
	K212A_FD	gaggaaggcgtaaccttcgcaatcggctctggattctc	
Psy-lig S215A	S215A_BK	ctcatcggagaatccagcgccgattttgaaggtta	
	S215A_FD	taaccttcaaaatcggcgctggattctccgatgag	
Psy-lig K25A	K25A_FD	cgcacgaattccatcaagtgcctcactcacgtagtactgt	
	K25A_BK	acagtactacgtgagtgaggcacttgatggaattcgtgcg	
Psy-lig R80A	R80A_corr_FD	ctgattatcctgcgtggcagcaatgccgctaacg	
	R80A_corr_BK	cgttagcggcattgctgccacgcaggataatcag	
Psy-lig N144A	N144A_FD	ttgcccatgacgaccttggccagtaagtcgaacagtgc	
	N144A_BK	gcactgttcgacttactggccaaggtcgtcatgggcaa	
Psy-lig K176A	K176_FD	tcgcttcggcatcctcaaatgccttcagtttcatcagatcac	
	K176_BK	gtgatctgatgaaactgaaggcatttgaggatgccgaagcga	
Ame-Lig D-Loop	Ama_5'_fragment_FD	gctcgggccccaaataat	

	Ama_5’_fragment_BK	accggatccaacaatgcttgcgg	
	Ama_3’_fragment_FD	gcattgttggatccggtgcaac	
	Ama_3’_fragment_BK	cgcagaaagcttgcaaaacg	
Nicked duplex for crystallization	Oligo1_Xtal_21_5P	cac tat cgg aa	5’ Phosphate
	Oligo_2Xtal_21_ 3OH	att gcg acc	
	Oligo_3Xtal_21 _compl	ttc cga tag tgg ggt cgc aat	
MST / EMSA assay	MST_5-prime_nick	tccgaattcgagctccgtcg	5’ Phosphate
	MST_3-prime_nick	aggccatggctgatatcgga	5’ FAM (6-Carboxyfluorescein)
	EMSA_linear	aggccatggctgatatcggatccgaattcgagctccgtcg	
	MST_EMSA_Complement	cgacggagctcgaattcggatccgatatcagccatggcct	

Sup. Table 2

Supplementary Table 2. List of Gene identifiers used to make sequence logo in Figure 2D. Lig Es were identified by downloading all sequences in the NCBI non-redundant database possessing only pfam domains 01068 and 14743 and no other large N- or C-terminal extensions (greater than 40 amino acids). Only sequences from bacteria were considered, and sequences were further curated by removing those with insert regions corresponding to the Chrlorella Virus latch.

108762460:1, 109698685:2, 110648674:2, 113461467:2, 113889205:2, 114551501:0, 114562661:1, 119767630:1, 119899420:2, 120326449:1, 120559174:2, 120593889:2, 121548580:0, 121555068:1, 121612332:1, 124115978:0, 124121829:0, 124259327:1, 125997283:1, 126630889:0, 127512516:1, 144979615:0, 144986314:0, 145269720:0, 145274058:0, 145845815:0, 145964533:1, 148836184:0, 148871386:0, 148874515:0, 149358125:0, 150423565:0, 151937452:3, 152939420:2, 152978858:1, 153000363:1, 154148219:1, 154174003:1, 157847332:2, 159175300:1, 160875001:1, 161327552:0, 161594697:1, 167623672:1, 170718422:1, 170776838:1, 197334485:2, 207085038:1, 212557346:1, 217505866:2, 218322528:1, 218767380:1, 219549110:1, 222538303:2, 222878442:0, 223693030:2, 224942385:0, 224953110:0, 229333193:0, 229337210:0, 229342399:0, 229343247:0, 229347650:0, 229375171:0, 237865724:0, 239522875:0, 240296784:0, 241320825:0, 241366113:0, 24373759:2, 251792811:1, 253511228:1, 254667684:2, 254671206:0, 254673752:0, 255050284:0, 255299477:0, 257048132:2, 257441177:0, 257450691:0, 258519633:0, 260092246:0, 260094744:0, 260221552:0, 260604903:0, 260611937:0, 260616419:0, 260631813:0, 261310137:0, 261312662:0, 262219625:0, 268161204:0, 268586712:0, 268625894:0, 268680415:1, 269145004:0, 269826815:0, 269832545:0, 283791555:0, 283794757:0, 288566625:0, 288895391:1, 291309676:0, 294340788:2, 296838385:0, 297258919:1, 297536704:0, 298282314:0, 304337340:0, 307630526:1, 308274374:0, 309370727:0, 309751174:1, 309973353:1, 311696348:1, 313005086:1, 313128330:0, 315014568:2, 315477350:0, 315479499:0, 315480589:0, 317432855:2, 319786663:1, 319793503:1, 319999433:3, 322118447:0, 323313079:0, 323315918:0, 325127326:0, 325129366:0, 325135279:0, 325143605:0, 325159671:1, 325201096:2, 327464770:0, 327467512:0, 327484167:1, 328492291:0, 329310973:2, 330722467:0, 332038463:0, 332040004:0, 332110909:0, 332975175:0, 333442403:0, 333477997:1, 333788494:0, 337256912:2, 339885800:0, 340046316:0, 340553674:2, 341949603:0, 341950773:0, 341952540:0, 341954193:1, 341956210:0, 342327970:0, 342793473:0, 342794915:0, 342800104:1, 342803026:0, 342814006:1, 343393084:0, 343922740:0, 343968627:0, 343970757:0, 344223456:0, 345124272:0, 345429798:2, 345471252:2, 347814584:0, 347992773:0, 348010987:0, 348013602:0, 348030192:2, 348609746:0, 348654560:0, 349795096:0, 356447824:0, 356485893:0, 357224757:0, 357418692:1, 359355653:1, 359755462:0, 363417033:0, 363650660:0, 364577889:0, 371494272:0, 371758385:0, 374569211:0, 375130926:1, 37679818:3, 380518440:0, 380522746:0, 380524289:0, 380547839:0, 380562183:0, 380576237:0, 380598228:0, 380605437:0, 380618743:0, 380625359:0, 380631125:0, 380631893:0, 380644329:0, 380646912:0, 380648026:0, 380653481:0, 380657079:0, 380660095:0, 380676879:0, 380833337:0, 381341012:1, 383759117:2, 384155360:1, 384578144:0, 385193162:0, 385280912:1, 385695510:0, 385697466:0, 386313535:1, 386324941:2, 386762450:1, 386903442:0, 386905425:0, 386909012:0, 389402845:0, 389606800:0, 390427927:1, 390941000:2, 391859872:0, 392605313:0, 395940738:0, 395951730:0, 395953387:0, 396087060:0, 398085940:1, 398096489:0, 400377745:0, 402314832:0, 402315133:0, 402320923:0, 402328990:0, 402334157:0, 402339080:0, 402342830:0, 406595607:2, 407207921:0, 407254106:1, 407289846:1, 407717391:2, 407766070:0, 408024741:0, 408032471:0, 408876000:0, 408886029:1, 408897334:0, 410817639:1, 421960779:0, 429161732:0, 429227365:0, 432200730:0, 432245331:0, 441428075:0, 444242377:0, 444507245:0, 446831227:1, 446831232:1, 451931819:0, 452086336:0, 459278227:0, 469611768:0, 469675993:0, 469740180:0, 473828414:1, 476415868:2, 478443157:0, 478770947:0, 479290958:0, 479293810:1, 479298890:0, 479299892:0, 479304517:0, 485369548:0, 485371702:0, 487813709:1, 488493776:1, 488958630:0, 489014976:1, 493693947:1, 493844358:1, 494254078:1, 494855212:0, 494922432:1, 495384871:0, 495390444:1, 495402835:0, 495409996:0, 495740102:0, 496174095:1, 496439758:1, 497235787:1, 499159468:0, 499540372:3, 499581254:2, 499942220:2, 500036612:2, 500129912:2, 500198005:2, 500863814:2, 501017387:2, 501509354:2, 501545495:2, 501583519:2, 502270739:2, 502816860:2, 503284845:2, 503337192:1, 503426423:2, 503511113:2, 503518072:2, 503622783:2, 503916277:2, 503997788:2, 504058922:2, 504387185:2, 504517458:2, 504519037:2, 504685824:2, 504810245:2, 504827579:2, 505245903:2, 508731256:0, 511531095:1, 514068023:0, 514213579:0, 519121900:0, 519729731:0, 519734778:0, 521321957:2, 521331215:1, 522588844:1, 52424994:3, 525951466:2, 526460051:1, 527845967:0, 528051668:0, 528865392:0, 529063827:1, 530446374:1, 530900685:0, 530920525:0, 531075339:0, 532689335:1, 536749218:0, 537385543:2, 540223626:0, 540254565:0, 540265980:0, 540275439:0, 540287581:0, 540291155:0, 540299115:0, 541484295:0, 543112574:0, 543203743:0, 543204697:0, 543206092:0, 543210800:0, 543211050:0, 543212186:0, 543942448:2, 543997526:2, 544270561:1, 545092018:2, 548710279:0, 548719101:0, 550663513:0, 550669293:0, 550678117:0, 550687597:0, 550784566:0, 553946525:0, 556854727:2, 556909054:0, 564137010:0, 56461154:2, 564723455:0, 564726596:0, 56479461:2, 566042285:1, 566128547:1, 567328127:0, 567931867:1, 57016433:0, 570731467:0, 572102750:0, 575442715:0, 575444850:0, 575448172:0, 575449292:0, 575455037:0, 575466035:0, 584422161:0, 584427168:0, 586630466:0, 589590181:0, 589833550:0, 589849863:0, 589862263:0, 589867941:0, 594554674:0, 597958196:0, 59802342:1, 617571959:3, 631603118:0, 631603948:0, 631604902:0, 631607056:0, 631610171:0, 631613891:0, 631614913:0, 631619493:0, 631621339:0, 631621378:0, 633290206:0, 633467255:0, 633468462:1, 636861769:4, 636864101:1, 639233760:1, 643595045:1, 643751368:1, 644637549:1, 646230529:0, 653289362:0, 653290695:0, 653655435:0, 655610060:0, 655645008:0, 655679620:2, 655774772:15, 656105254:0, 656243206:1, 657186254:0, 657846125:3, 663090443:0, 666933711:0, 667804942:0, 667808175:1, 668679266:1, 668682794:1, 669188772:1, 669349005:1, 669354371:1, 669379151:2, 669383382:2, 669403387:0, 669407186:0, 669636420:0, 672593466:0, 672869419:0, 673469809:1, 673920474:0, 674436663:2, 674451032:3, 674769555:1, 68057928:2, 683414620:1, 686988878:0, 691217845:0, 692306388:0, 693433827:1, 693595283:0, 693736443:1, 694143563:1, 694336114:0, 694338135:0, 694342493:0, 694346809:0, 694866868:2, 695267158:1, 696159977:1, 696169936:1, 696250674:1, 699263904:0, 699270081:0, 700286800:1, 700655470:0, 702394408:0, 702396891:1, 702411633:0, 703561754:2, 703566574:0, 703605353:3, 703612352:0, 703618279:0, 704010822:0, 706089265:1, 71147864:1, 71906874:1, 719383128:1, 720062506:1, 722085810:1, 723632602:1, 725529600:0, 736316772:1, 736609839:1, 73696000:0, 737250863:1, 737480016:1, 738610062:1, 739113611:1, 740373965:1, 74056325:1, 745807338:0, 746003614:1, 746012724:1, 746014644:1, 746020635:1, 746067125:1, 746092098:1, 746104999:1, 746401036:1, 746420514:1, 747184865:11, 76876011:1, 78486013:2, 78497323:1, 81353134:2, 84379502:0, 85836232:0, 85840703:0, 86162400:0, 88817562:0, 89900229:1, 90327823:0, 90441034:0, 91191251:0, 91788281:2, 94426272:1,

Sup. Table 3

Supplementary Table 3. Functional conservation of interactions between the linker region and catalytic domain in a selection of Lig E proteins. For details of interactions see Figure 5 of main text. Name abbreviations are: Hin-lig, Haemophilus influenzae; Nme-lig, Neisseria meningitidis; Vch-lig, Vibrio cholera; Vib-lig, Aliivibrio salomicida.

Protein	**Polar interactions**					**Hydrophobic interactions**			
	N144 - K176		K176 - E152	E178-N10					
Psy-Lig	N144	K176	E152	E178	N10	Y11	F140	F177	F247
Ama-Lig	E	Q	E	E	Q	Y	E	F	F
Hin-Lig	A	T	E	R	T	Y	Y	A	F
Nme-Lig	K	S	E	Y	E	Y	M	Q	F
Vch-Lig	D	R	E	Q	N	Y	L	H	F
Vib-Lig	E	K	E	Y	S	Y	L	H	F
Par-Lig	E	P	E	L	V	Y	T	Y	F
